# Preparation of single-site tin(IV) compounds and their use in the polymerization of ε-caprolactone

**DOI:** 10.1080/15685551.2016.1231032

**Published:** 2016-09-26

**Authors:** Begum Canan Yildiz, Asgar Kayan

**Affiliations:** ^a^ Department of Chemistry, Kocaeli University, Kocaeli, Turkey

**Keywords:** Single-site tin catalyst, carboxylates, ε-caplolactone, ring-opening, NMR

## Abstract

Butyltin(IV) carboxylate compounds were obtained by reactions of butyltrichlorotin(IV) with potassium pivalate, perfluoroheptanoate, methacrylate, 2,6-pyridinedicarboxylate, and phthalate. The synthesized complexes were fully characterized by nuclear magnetic resonance (^1^H-, ^13^C-NMR), Fourier transform infrared (FTIR), mass spectroscopies (MS) and elemental analysis. These tin complexes were used as catalysts for the ring opening polymerization of ε-caplolactone and the conversion of monomers to polymers was completed in just 1 h. The structures of polymers were characterized by a combination of spectroscopic techniques (NMR, FTIR, MS), differential scanning calorimeter (DSC) and gel permeation chromatography. In this study, the ε-caplolactone polymers with different average molecular weights between 5000 and 40,000 Da having a regular structure were obtained.

## 1. Introduction

Organotin(IV) derivatives show variety of applications ranging in all sorts of biological activities as anticancer, antiviral, antibacterial and antifungal agents, wood preservatives, pesticides, etc., precursors for hybrid inorganic–organic nano and non-linear optical materials, catalysts for organic carbon–carbon coupling and ring opening processes.[1–5] Despite their broad utilities, it is surprising that there is very little information about the synthesis of single-site organotin derivatives and their usage as catalysts in the ring opening of cyclic esters such as ε-caprolactone or L-lactide.[6,7] The single-site catalyst has a general formula of L_n_RMX (M: Metal, R: alkyl or aryl). The steric and electronic properties of the ancillary ligands (L_n_) adjust the bonding of the metal center to the ligands and influence the activity and stereo-selectivity of the catalysts. The initiating group, X, affects the polymerization activity of the compounds.[8–10] Therefore, it is important to synthesize new organotin derivatives by appropriate combinations of L_n_ and X to produce efficient catalysts which can precisely control the polymerization rate, molecular weight and polymer stereo-chemistry in polymerization reactions. In order to see their catalytic activity in the ring opening polymerization (ROP) of cyclic esters, ε-caprolactone or lactide was chosen as a monomer because of their applications in versatile areas such as drug delivery systems, medical devices, dentistry, bone and tissue engineerings.[11,12] Poly-ε-caprolactones (PCL) is a hydrophobic and semi-crystalline polymer which has good solubility, low melting point (59–64 °C) and exceptional blend-compatibility. These properties have stimulated extensive research into its potential application in the medical field.[11–13]

Firstly in this context, the synthesis and characterization of a family of single-site butyltin(IV) pivalate, perfluoroheptanoate, methacrylate, phthalate, and 2,6-pyridinedicarcoxylate compounds were reported. Secondly, the study of the title compounds in the ROP of ε-caprolactone was included. Finally, the obtained PCL were fully characterized by Fourier transform infrared (FTIR), ^1^H-NMR, ^13^C-NMR, mass spectroscopies (MS) and gel permeation chromatography (GPC).

## 2. Experimental

### 2.1. Materials and instruments

Butyltrichlorotin(IV) (BuSnCl_3_, 95%, Alfa Aesar), potassium hydroxide reagent (KOH, 85% min, Merck), pivalic acid (PivH, 99%, Merck), methacrylic acid (MAcH, 98%, Fluka), perfluoroheptanoic acid (PFHH, 99%, Sigma), phthalic acid (PHH, 100%, Merck), 2,6-pyridinedicarboxylic acid, (PydH, 99%, Aldrich), ε-caprolactone (ε-CL, 99%, Alfa Aesar) were used as received. Ethanol (99.8%, Sigma-Aldrich) and toluene (99.7% Sigma-Aldrich) were dried over activated 4Å molecular sieves before use.

The infrared spectra of butyltin(IV) compounds, potassium carboxylates and PCL were recorded on a Brucker Tensor 27 FTIR spectrophotometer using single reflection ATR universal plate of diamond crystal. The K-, Sn-compounds, and PCL were scanned from 400 to 4000 cm^−1^ with a resolution of 4 cm^−1^. The elemental analysis was carried out with the Costech ECS 4010 CHNS-O elemental analyzer. Differential scanning calorimeter (DSC, Metler Toledo DSC 1 Star System) analysis was performed to obtain thermal properties of PCL. The films were heated from −10 to 100 °C at a heating rate of 10 °C/min under a nitrogen gas in order to prevent oxidative degradation. GPC analysis was performed at 30 °C on a Shimadzu prominence GPC system equipped with a RID-10A refractive index detector, a LC-20AD solvent delivery unit, a CTO-10AS column oven and a set of two columns, PSS SDV 5 μL 1000 Å and PSS SDV 5 μL 50 Å. THF (HPLC grade) was used as the mobile phase at 1.0 mL/min. The sample concentration was 2 mg/mL and the injection volume was 50 μL. The calibration curve was made with polystyrene standards covering the molecular weight range from 162 to 67,000 Da.

## 3. Preparation of potassium carboxylates

### 3.1. Preparation of potassium phthalate

Phthalic acid (6.0 mmol, 1.0 g) was added to the solution of KOH (12.0 mmol, 0.79 g) in 15 ml distilled water. The reaction was stirred at RT for 30 min and then the solvent water was removed at 65 °C by vacuum evaporator. The white solid was washed two times with ethanol and one time with toluene and dried under vacuum. Elemental analysis, C_8_H_4_O_4_K_2_, Found: C, 38.85; H, 1.60%. Calculated: C, 39.65; H, 1.66%. FTIR (cm^−1^): 3017, 2970, 2934, 2870, 1635, 1608, 1575, 1429, 1368, 1072, 999, 901, 814, 752, 719, 650, 406.

The preparation methods for K_2_-2,6-pyridinedicarboxylate, K-methacrylate, K-pivalate, and K-perfluoroheptanoate were similar to that of K_2_-phthalate except that the amount of KOH was different. Similarly, K_2_-2,6-pyridinedicarboxylate was washed two times with ethanol and one time with toluene and dried under vacuum at 65 °C overnight. In contrast to K_2_-phthalae and K_2_-2,6-pyridinedicarboxylate, K-methacrylate, K-pivalate, and K-perfluoroheptanoate were not washed with toluene because they were more soluble in toluene. These potassium carboxylates were prepared similar to literature methods.[14,15]

## 4. Preparation of butyltin(IV) carboxylates

### 4.1. Preparation of butylchlorobis(pivalato)tin(IV) compound

$$2{\left({\text{CH}}_{3}\right)}_{3}\text{CCOOK}+{\text{BuSnCl}}_{3}⟶1/2{\left[\text{Bu}{\left({\left({\text{CH}}_{3}\right)}_{3}\text{CCOO}\right)}_{2}\text{SnCl}\right]}_{2}$$

Potassium pivalate (1.68 g, 12.0 mmol) was added to the solution of butyltrichlorotin (1.79 g, 6.0 mmol) in 30 ml of ethanol and then solution was refluxed for 3 h. After refluxing, the solvents were removed under vacuum at 50 °C to give a colorless liquid. To remove KCl, the liquid product was dissolved in ~20 ml of toluene and then the white solid was removed by filtration. The solvent toluene was removed from filtrate under vacuum at 65 °C to give colorless liquid product. Elemental analysis: (C_28_H_54_Cl_2_O_8_Sn_2_, *M*_*w*_ = 827.04 g/mol) Calculated: C, 40.66; H, 6.58%. Found: C, 40.10; H, 6.40%. TOF MS ES+(solvent-ethanol): 873.05 Da [((CH_3_)_3_CCOO)_4_Sn_2_Cl_2_(C_4_H_9_)_2_ + C_2_H_5_OH + H]^+^, 756.96 Da [((CH_3_)_3_CCOO)_4_Sn_2_(C_4_H_9_)_2_ + H]^+^, 538.93 Da [((CH_3_)_3_CCOO)_2_Sn_2_(C_4_H_9_)(CH_2_CH_2_CH) + H]^+^ . ^1^H-NMR, CDCl_3_, ppm, *δ*: 0.94 (t, 6H, δ-CH_3_), 1.21 (s, 36H, C(CH_3_)_3_), 1.45 (m, 4H, *γ*-CH_2_), 1.69 (m, 4H, *β*-CH_2_), 1.83 (t, 4H, *α*-CH_2_). ^13^C-NMR, CDCI_3_, ppm, *δ*: 13.64 (CH_3_), 25.52 (γ-CH_2_), 27.11 (β-CH_2_), 27.19 (α-CH_2_), 27.41 (*C*(CH_3_)_3_), 188.3 (COO terminal-bidentate), 186.9 (COO bridging-bidentate) . FTIR (cm^−1^): 2965, 2930, 2870, 1553, 1484, 1423, 1371, 1227, 880, 786, 686. [Sn-^*α*^CH_2_^*β*^CH_2_^*γ*^CH_2_^*δ*^CH_3_].

### 4.2. Preparation of butylchlorobis(methacrylato)tin(IV) compound

$$2{\text{CH}}_{2}=\text{C}({\text{CH}}_{3})\text{COOK}+{\text{BuSnCl}}_{3}⟶1/2[\text{Bu}({\text{CH}}_{2}=\text{C}({\text{CH}}_{3})\text{COO}{)}_{2}\text{SnCl}{]}_{2}$$

By a similar procedure to 4.1, potassium methacrylate (0.37 g, 3.0 mmol) and butyltrichlorotin (0.44 g, 1.5 mmol) gave [Bu(CH_2_=C(CH_3_)COO)_2_SnCl]_2_ as a colorless liquid. Elemental analysis (C_24_H_38_Cl_2_O_8_Sn_2_ *M*_*w*_ = 762.88 g/mol) Calculated: C, 37.79; H, 5.02%. Found: C, 37.26; H, 5.04%. TOF MS ES+(solvent-ethanol): 801.0 Da [(CH_2_=C(CH_3_)COO)_4_Sn_2_Cl_2_(C_4_H_9_)_2_ + C_2_H_5_OH + H]^+^, 522.9 Da [(CH_2_=C(CH_3_)COO)_2_Sn_2_(C_4_H_9_)_2_ + H], 374.2 Da [(CH_2_=C(CH_3_)COO)_2_Sn_2_(C_4_H_9_) (CH_2_CH) + H]^+^. ^1^H-NMR, CDCI_3_, ppm, *δ*: 0.93 (t, 6H, *δ*-CH_3_), 1.25 (m, 4H, *γ*-CH_2_), 1.43 (m, 4H, *β*-CH_2_), 1.82 (t, 4H, *α*-CH_2_), 1.94 (s, 12H, =CCH_3_), 5.68 (d, 2H, =CH_2_), 6.25 (d, 2H, =CH_2_). ^13^C-NMR, CDCI_3_, ppm, *δ*: 13.58 (*δ*-CH_3_), 18.04 (*γ*-CH_2_), 18.18 (=C*C*H_3_) 25.57 (*β*-CH_2_), 27.00 (*α*-CH_2_), 128.35 (=CH_2_, bridging), 129.87 (=CH_2_, terminal), 136.30 (=*C*CH_3_, bridging), 137.54 (=*C*CH_3_, terminal), 174.46 (COO, bridging-bidentate), 175.88 (COO, terminal-bidentate). FTIR (cm^−1^): 2960, 2929, 2867, 1639 (C=C), 1543 (COO), 1453, 1413 (COO), 1242, 948, 827.

Under the same reaction condition, the product [(CH_2_=C(CH_3_)COO)_2_Sn_2_Cl_4_(C_4_H_9_)_2_(HOOCC(CH_3_)=CH_2_)_2_] formed when methacrylic acid was used as a ligand instead of potassium methacrylate. TOF MS ES+(solvent-ethanol): 862.87, 864.87, 866.87 Da [(CH_2_=C(CH_3_)COO)_2_Sn_2_Cl_4_(C_4_H_9_)_2_(HOOCC(CH_3_)=CH_2_)_2_ + C_2_H_5_OH]^+^.

### 4.3. Preparation of butylchlorobis(perfluoroheptanoato)tin(IV) compound

$$2{\text{CF}}_{3}{\left({\text{CF}}_{2}\right)}_{5}\text{COOK}+{\text{BuSnCl}}_{3}⟶\left[\text{Bu}{\left({\text{CF}}_{3}{\left({\text{CF}}_{2}\right)}_{5}\text{COO}\right)}_{2}\text{SnCl}\right]$$

By a similar procedure to 4.1, potassium perflouroheptanoate (1.21 g, 3.0 mmol) and butyltrichlorotin (0.44 g, 1.5 mmol) gave [Bu(CF_3_(CF_2_)_5_COO)_2_SnCl] as a colorless liquid.

Elemental analysis: (C_18_H_9_ClF_26_O_4_Sn, *M*_*w*_ = 937.38 g/mol) Calculated: C, 23.06; H, 0.97%. Found: C, 22.36; H, 1.22%. TOF MS ES+(solvent-ethanol): 938.9 Da [(C_6_F_13_COO)_2_Sn(C_4_H_9_)Cl + H]^+^, 500.9 Da [(C_6_F_11_COO)Sn(C_4_H_9_) + H]^+^. ^1^H-NMR, CDCI_3_, ppm, *δ*: 0.93 (t, *δ*-CH_3_), 1.29 (m, 2H, *γ*-CH_2_), 1.41 (m, 2H, *β*-CH_2_), 1.74 (t, 2H, *α*-CH_2_). ^13^C-NMR, CDCI_3_, ppm, *δ*: 13.29 (*δ*-CH_3_), 17.79 (*γ*-CH_2_), 25.43 (*β*-CH_2_), 26.87 (*α*-CH_2_), 108.52–118.27 (CF_2_), 188.10 (COO). FTIR (cm^−1^): 2967, 2936, 2879, 1650, 1429, 1360, 1233, 1200, 1143, 1050, 851, 806, 744, 719, 664.

Under the same reaction condition, the product [(C_6_F_13_COO)Sn(C_4_H_9_)Cl_2_(HOOCF_13_C_6_).H_2_O + H]^+^ formed when perfluoroheptanoic acid was used as a ligand instead of potassium perfluoroheptanoate. TOF MS ES+: 990.84, 992.84, 994.84 Da [(C_6_F_13_COO)Sn(C_4_H_9_)Cl_2_(HOOCF_13_C_6_).H_2_O + H]^+^, 972.84, 974.84, Da[(C_6_F_13_COO)Sn(C_4_H_9_)Cl_2_(HOOCF_13_C_6_) + H]^+^.

### 4.4. Preparation of butylchloro(2,6-pyridinedicarboxylato)tin(IV) compound

$${\text{C}}_{5}{\text{H}}_{3}\text{N}{\left(\text{COOK}\right)}_{2}+{\text{BuSnCl}}_{3}⟶1/2{\left[\text{Bu}\left({\text{C}}_{5}{\text{H}}_{3}\text{N}{\left(\text{COO}\right)}_{2}\right)\text{SnCl}\right]}_{2}$$

Potassium 2,6-pyridinedicarboxylate (0.73 g, 3.0 mmol) was added to the solution of butyltrichlorotin (0.89 g, 3.0 mmol) in 30 ml of ethanol and then solution was refluxed for 3 h. After refluxing, the solvents were removed from mixture under vacuum at 50 °C. To remove KCl from mixture, the mixture was dissolved in ~5 ml of water and precipitated with ~25 ml of ethanol. The soluble parts including KCl were removed by decantation and then the white solid product was re-washed two times with ethanol and dried at 65 °C under reduced vacuum. Elemental analysis (C_22_H_24_N_2_O_8_Sn_2_Cl_2_, *M*_*w*_ = 752.76 g/mol): Calculated: C, 35.10; H, 3.21; N, 3.72%. Found: C, 34.36; H, 3.41; N, 3.63%. TOF MS ES+(solvent-ethanol): 497.2 Da [C_11_H_12_NO_4_ClSn_2_ + H]^+^, 474.9, 457.9, 441.0, 409.9 Da. ^1^H-NMR, CDCI_3_, ppm, *δ*: 0.93 (t, 6H, *δ*-CH_3_), 1.25 (m, 4H, *γ*-CH_2_), 1.43 (m, 4H, *β*-CH_2_), 1.82 (t, 4H, *α*-CH_2_), 8.50 (d, 4H, C_5_H_3_ N), 8.70 (t, 2H, C_5_H_3_ N) . ^13^C-NMR (DMSO) *δ*: 162 (COO), 147, 141, 128 (C_5_H_3_), 38 (*α*-CH_2_), 28 (*β*-CH_2_), 26 (*γ*-CH_2_), 13 (*δ*-CH_3_). FTIR (cm^−1^): 2961, 2930, 2872, 1676 (C=O), 1618 (C=C), 1578 (C=N), 1398, 1341, 1173, 1082, 1043, 919, 878, 743, 681.

### 4.5. Preparation of butylchloro(phthalato)tin(IV) compound

$${\text{C}}_{6}{\text{H}}_{4}{\left(\text{COOK}\right)}_{2}+{\text{BuSnCl}}_{3}⟶\left[\text{Bu}\left({\text{C}}_{6}{\text{H}}_{4}{\left(\text{COO}\right)}_{2}\right)\text{SnCl}\right]$$

By a similar procedure to 4.4, potassium phthalate (0.73 g, 3.0 mmol) and butyltrichlorotin (0.89 g, 3.0 mmol) produced [Bu(C_6_H_4_(COO)_2_)SnCl] as a white solid. Elemental analysis (C_12_H_13_ClO_4_Sn, *M*_*w*_ = 375.4 g/mol): Calculated: 38.39 C, H 3.49%. Found: C 37.40, H 3.58%. TOF MS ES+(solvent-ethanol): 376.9 Da [C_12_H_13_ClO_4_Sn + H]^+^. ^1^H-NMR, CDCI_3_, ppm, *δ*: 0.82 (t, 3H, *δ*-CH_3_), 1.10 (m, 4H, *γ*-CH_2_, *β*-CH_2_), 1.35 (t, 2H, *α*-CH_2_), 7.4 (d, 2H, C_6_H_4_), 7.5 (d, 2H, C_6_H_4_). ^13^C-NMR (DMSO) *δ*: 169 (COO), 133, 130, 128, 127 (C_6_H_4_), 28 (*α*-CH_2_), 26 (*β*-CH_2_), 25 (*γ*-CH_2_), 13 (*δ*-CH_3_).FTIR (cm^−1^): 2957, 2928, 2870, 1710, 1548, 1403, 1081, 750, 695, 472.

## 5. Polymerization of ε-caprolactone with butyltin carboxylates

The catalyst ([Bu(C_4_H_5_O_2_)_2_SnCl]_2_, 15 mg) was mixed with ε-caprolactone (1.4 mL) in a vial under nitrogen atmosphere. The solvent free mixture was stirred at 80 °C for 1 h. GPC results: *M*_*w*_ = 29,600 Da., *M*_*n*_ = 26,510 Da., *M*_*w*_/*M*_*n*_ = 1.12. ^1^H NMR, CDCl_3_, ppm, *δ*: 4.05 (t, *J* = 7.0 Hz, ^ε^CH_2_-O), 2.30 (t, *J* = 7.0 Hz, ^*α*^CH_2_–C=O), 1.64 (m, ^*β,δ*^CH_2_), 1.37 (m, ^*γ*^CH_2_). ^13^C NMR, CDCl_3_, ppm, *δ*: 173.6 (C=O), 64.2 (^*ε*^CH_2_O), 34.1 (^*α*^CH_2_), 28.3 (^*δ*^CH_2_), 25.5 (^*β*^CH_2_), 24.6 (^*γ*^CH_2_). FTIR (cm^−1^): 2943 (CH_2_, asym str), 2865 (CH_2_, sym str), 1722 (C=O), 1471 (CH_2_, bending), 1367 (CH_2_, bending), 1293 (C–C), 1241 (C–O–C, asym), 1167 (C–O–C, sym), 1047, 962, 732. [O=C–^*α*^CH_2_^*β*^CH_2_^*γ*^CH_2_^*δ*^CH_2_^*ε*^CH_2_O–]

The polymerization reactions of ε-caprolactone with other butyltin(IV) carboxylates were carried out the under similar conditions to the reaction above. They were also very effective in the ring opening of ε-caprolactone.

## 6. Results and discussion

**Scheme 1.**
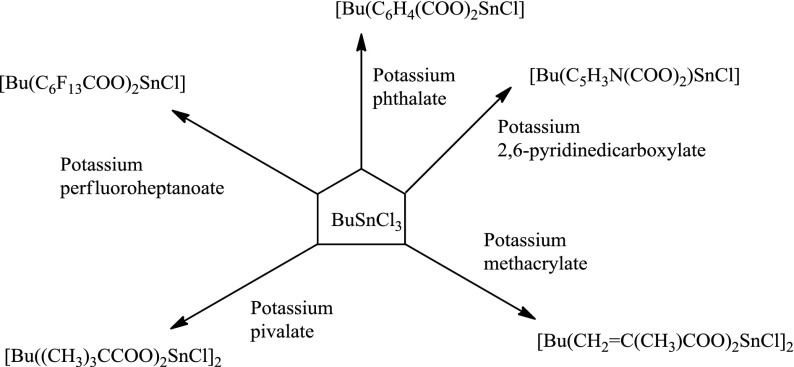
Reactions of BuSnCl_3_ with different carboxylate salts.

As mentioned in the experimental section, firstly potassium salts of phthalate, 2,6-pyridinedicarboxylate, methacrylate, pivalate, and perfluoroheptanoate were prepared as in literatures.[14–16] The known structures of these potassium carboxylates were confirmed by elemental analysis and FTIR measurements. Secondly, the single-site tin compounds described in this study were prepared by ligand substitution reactions as shown in Scheme 1.

To our knowledge, tin pivalate, methacrylate, and perfluoroheptanoate compounds in these compositions were synthesized for the first time and therefore, they should be fully characterized. The structures of these single-site tin(IV) compounds were characterized using a combination of elemental analysis and spectroscopic techniques such as ^1^H-, ^13^C-NMR, FTIR, and MS. Colorless liquid monocarboxylate-tin compounds and white solid dicarboxylate-tin compounds were relatively air and moisture stable. Their elemental analysis results were in good agreement with the proposed formulations as shown in Scheme 1.

The FTIR absorption bands provided information about the formation of tin-carboxylate compounds and the mode of coordination of each carboxylate ligand in its compounds. The peaks of all potassium and tin carboxylates shown in the FTIR spectra belonged to the coordinated carboxyl groups not the free carboxylic acid groups. For example, the FTIR spectrum of free pivalic acid exhibited intense band at 1697 cm^−1^ corresponding to asymmetrical stretching vibrations of the carboxyl group.[17] After the reaction of pivalic acid with KOH, the band shifted the low wave numbers ~1542 for νCOO_asym_ and ~1360 cm^−1^ for νCOO_sym_. As it was expected, FTIR spectrum of tin(IV) pivalate compound showed peaks at different wave numbers and regions. In the FTIR spectrum of tin(IV) pivalate compound, the bands at ~1553 and 1371 cm^−1^ indicated the bonding of carboxylate group as bidentate chelating mode. These values are consistent with those detected in a number of carboxylate–metal compounds.[18]

The ^1^H NMR spectra of all tin compounds exhibited signals corresponding to the organic-carboxylate groups as well as those of butyl group attached to the tin(IV) atom. The ^1^H-NMR spectrum of [Bu((CH_3_)_3_CCOO)_2_SnCl]_2_ showed peaks 0.94 (t, 6H, *δ*-CH_3_), 1.45 (m, 4H, *γ*-CH_2_), 1.69 (m, 4H, *β*-CH_2_), 1.83 (t, 4H, *α*-CH_2_) ppm for n-butyl group and at 1.21 (s, 36H, C(CH_3_)_3_) ppm for tert-butyl group. The ^13^C NMR spectrum of [Bu((CH_3_)_3_CCOO)_2_SnCl]_2_ showed four resonances at 13.64 (*δ*-CH_3_), 25.52 (*γ*-CH_2_), 27.11 (*β*-CH_2_), 27.19 (*α*-CH_2_) for *n*-butyl groups and three resonances at 27.41 (C(*C*H_3_)_3_), 41.7 (*C*(CH_3_)_3_), 189.41 (COO) for pivalate groups. The COOH resonance of pivalic acid at 179 ppm shifted down field to 188.3 and 186.9 ppm in the tin pivalate compound which indicated coordination of COO groups in two modes as bridging-bidentate and terminal-bidentate to the tin atoms.

**Figure 1.**
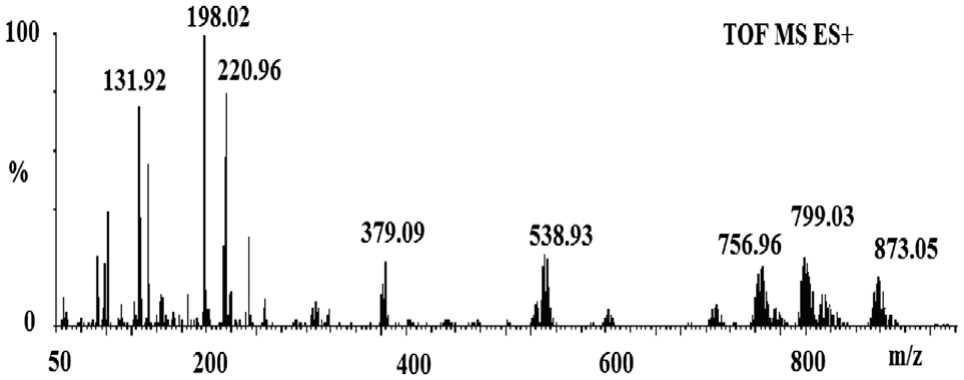
Mass spectrum of [Bu((CH_3_)_3_CCOO)_2_SnCl]_2_.

**Figure 2.**
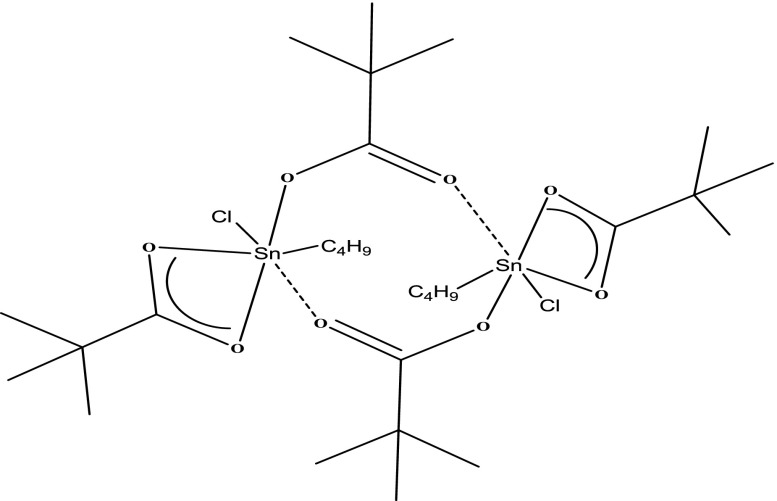
The dimeric structure of [Bu((CH_3_)_3_CCOO)_2_SnCl]_2_.

The MS measurements of all tin compounds were taken to prove the suggested formulations by elemental analysis, FTIR and NMR spectroscopies. The MS measurement showed that the reaction of potassium pivalate with butyltin trichloride yielded an dimeric compound [Bu((CH_3_)_3_CCOO)_2_SnCl]_2_ (Figure 1). The appearance of a peak at 873.05 Da for [C_28_H_54_Cl_2_O_8_Sn_2_ + C_2_H_5_OH + H]^+^ was a proof of the presence of dimeric structure as drawn in Figure 2.

MS measurements showed that while tin-methacrylate and tin-pivalate compounds were dimeric, the tin-perfluoroheptanoate compound was monomeric at 938.9 Da for [(C_6_F_13_COO)_2_Sn(C_4_H_9_)Cl + H]^+^. These kinds of tin carboxylate compounds are monomeric, dimeric or oligomeric depends on ligands. The dimeric or olygomeric compounds include bridging carboxylate ligands between two tin atoms.[19–21]

FTIR spectra of tin(IV) dicarboxylates were somewhat different from tin(IV) carboxylates. FTIR spectra of tin(IV) dicarboxylates showed that carboxylates group can be coordinated in two different modes at the same time such as bridging-bidentate and monodentate modes. For example, the FTIR spectrum of tin(IV) 2,6-pyridinedicarboxylate compound showed the band at 1676 cm^−1^ for monodentate binding (νC=O_asym_). This value is consistent with those detected in a number of carboxylate-tin complexes.[22] In the FTIR spectrum of [BuSnCl(OOC)_2_C_5_H_3_ N]_2_, the absence of brood ν(COOH) absorption in the range 3100–3400 cm^−1^ indicated a double deprotonation of the 2,6-pyridinedicarboxylic acid.

The ^1^H-NMR spectrum of [BuSnCl(OOC)_2_C_5_H_3_N]_2_ showed a doublet (4H, C_5_H_3_N) and a triplet (2H, C_5_H_3_N) of relative intensity 2:1 centered at 8.5 and 8.7 ppm which correspond to the protons of the 2,6-pyridinedicarboxylate groups. The other peaks in the ^1^H NMR spectrum at 0.94 (t, 6H, δ-CH_3_), 1.45 (m, 4H, *γ*-CH_2_), 1.69 (m, 4H, *β*-CH_2_), 1.83 (t, 4H, *α*-CH_2_) ppm belong to *n*-butyl groups. The ^13^C NMR spectrum of [BuSnCl(OOC)_2_C_5_H_3_N]_2_ showed four resonances at 13 (*δ*-CH_3_), 26(*γ*-CH_2_), 28 (*β*-CH_2_), 38 (*α*-CH_2_) ppm for *n*-butyl groups and four resonances at 128, 141, 147, and 162 (COO) ppm for 2,6-pyridinedicarboxylate groups. The COOH resonance of 2,6-pyridinedicarboxylic acid at 159 ppm was shifted down field to 162 ppm in the tin 2,6-pyridinedicarboxylate compound which indicated coordination of COO groups to the tin atoms.

**Figure 3.**
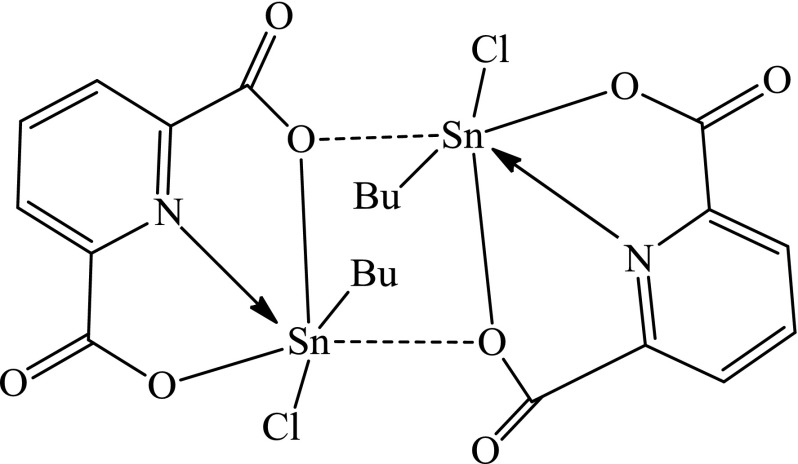
The dimeric structure of [BuSnCl(OOC)_2_C_5_H_3_N]_2_.

The MS measurement of tin-2,6-pyridinedicarboxylate compound showed that the reaction of potassium 2,6-pyridinedicarboxylate with butyltin trichloride was different from that of tin-phthalate compound. The reaction between potassium 2,6-pyridinedicarboxylate and butyltin trichloride yielded a dimeric compound [BuSnCl(OOC)_2_C_5_H_3_N]_2_. The appearance of a peak at 497.2 Da for [C_11_H_12_NO_4_ClSn_2_ + H]^+^ was a proof of the presence of dimeric structure as drawn in Figure 3.

The structural formula of [BuSnCl(OOC)_2_C_5_H_3_N]_2_ has shown similarities with the same compound prepared by different procedure.[23] In contrast to tin-2,6-pyridinedicarboxylate compound, MS measurement of tin-phthalate compound showed that this compound was monomer with molecular weight at 376.9 Da for [C_12_H_13_ClO_4_Sn + H]^+^.

In these tin compounds, chlorine atom was attached to the central tin atom. The presence of chlorine atom on butyltin(IV) moiety was an important factor to give activity to the complexes when they were used as catalysts in the ring opening of ε-caplolactone. The tin-carboxylate compounds were more active than tin-dicarboxylate compounds in the polymerization reactions. The tin-carboxylate compounds have aliphatic substituents (pivalate, methacrylate, and perfluoroheptonate); whereas tin-dicarboxylate compounds have aryl substituents (phthalate and 2,6-pyridinedicarboxylate).

**Figure 4.**
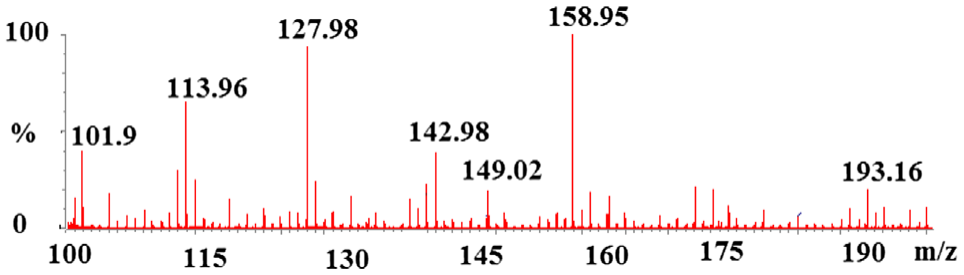
TOF MS ES+ spectrum of PCL prepared with [Bu(PFH)_2_SnCl].

The synthesized tin carboxylate compounds were used as catalysts in the polymerization reactions of ε-caprolactone and the obtained PCL were fully characterized by FTIR, ^1^H-NMR, ^13^C-NMR, MS and GPC. The chloride ligand on the tin catalyst was active group in the ring opening of ε-CL which was supported by mass data. MS measurements of PCL showed that chloride atom moved on to the ε-CL unite (the peak at 149.0 Da, PCL-Cl) (Figure 4). This MS measurement was performed after the first few minutes of polymerization reactions.

FTIR spectrum of PCL displayed characteristic peaks of C=O stretching vibrations at 1722 cm^−1^, CH_2_ bending modes at 1471 and 1367 cm^−1^ and CH_2_ asymmetric stretching at 2943 and symmetric stretching at 2865 cm^−1^. The C–O–C stretching vibrations gave peaks at 1047, 1167, and 1241 cm^−1^. The bands at 1167 and 1293 cm^−1^ were assigned to C–O and C–C stretching in the amorphous and in the crystalline phases, respectively.[24,25]

**Figure 5.**
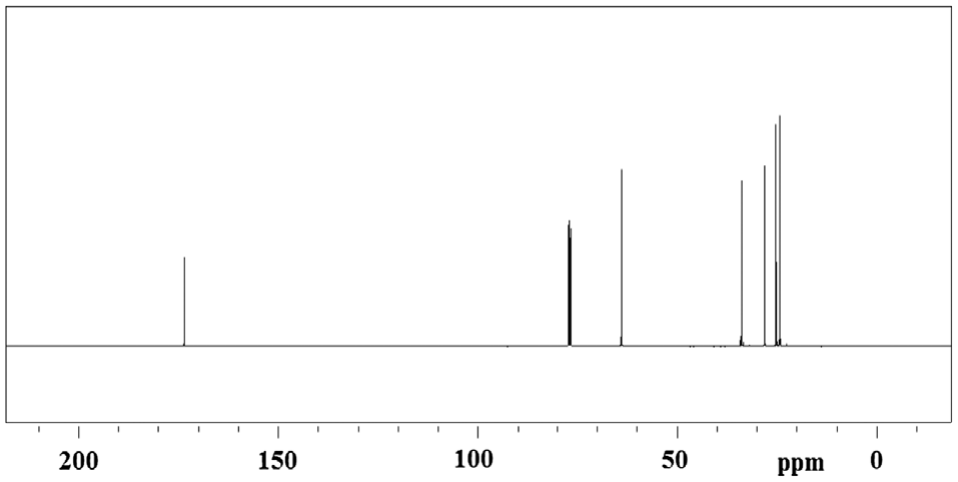
^13^C-NMR spectrum of PCL prepared with [Bu(PFH)_2_SnCl].

^13^C-NMR spectrum (Figure 5) of PCL showed six peaks that each carbon atom of PCL appeared at only one region 173.6 (C=O), 64.2 (^ε^CH_2_O), 34.1 (^*α*^CH_2_), 28.3 (^*δ*^CH_2_), 25.5 (^*β*^CH_2_), 24.6 (^*γ*^CH_2_) ppm. These data are the evidence of regular polymerization of ε-CL and are consistent with the literature data.[26]

**Figure 6.**
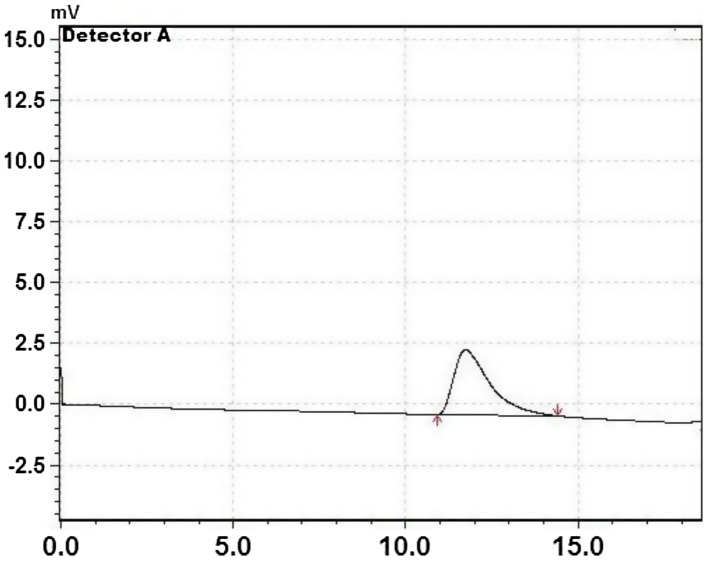
Gel permeation chromatogram of PCL prepared at 80 °C with the compound [Bu(PFH)_2_SnCl].

**Figure 7.**
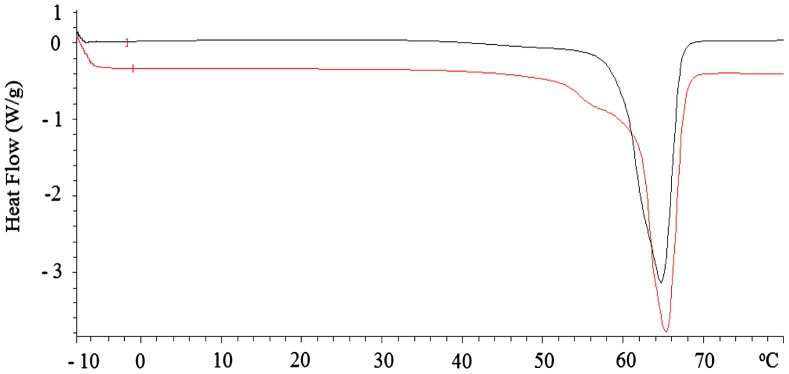
The heating DSC-thermogram of PCL at a heating rate of 10 °C/min. Red curve (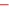): PCL prepared with [Bu(PFH)_2_SnCl], black curve (-): PCL prepared with [Bu(Piv)_2_SnCl]_2_.

**Scheme 2.**
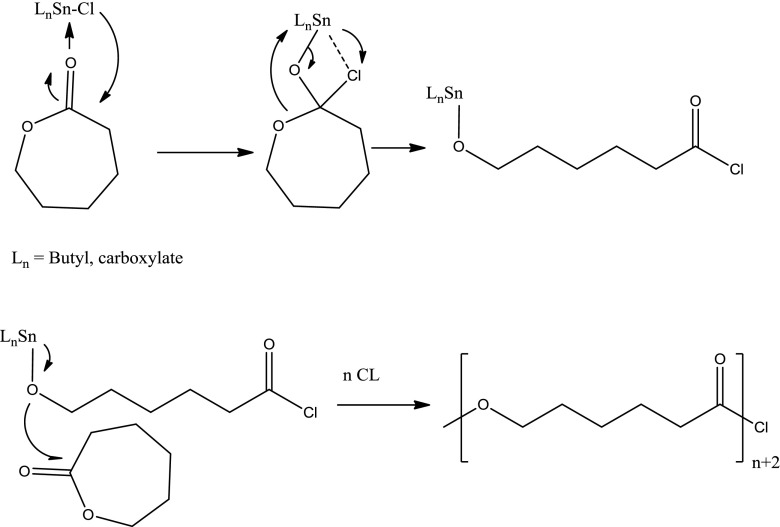
Proposed mechanism for the polymerization of ε-caprolactone by tin(IV) carboxylates.

These data suggest that first ε-caprolactone attacks to tin(Sn) center and then the nucleophile Cl^−^ ion attacks C=O carbon atom in the ε-caprolactone unit as seen in Scheme 2.[27] Then, lactone exocyclic oxygen coordinates to the tin atom. Propagation step involves successive ring-opening by an anionic coordination–insertion mechanism.[28]

GPC was also used to determine molecular weight and molecular weight distribution index of polymers. By varying the reaction times, temperatures and the catalysts, the CL polymers with different average molecular weights or number average molecular weights were obtained. These tin catalysts were very active for ε-CL polymerization at and above 60 °C. The polymerization of ε-CL was completed within 17 h at 60 °C. Therefore, polymerization reaction was accelerated by the increasing of temperature from 60 to 80 °C as seen in Table 1. For polymers of ε-CL prepared with [Bu(PFH)_2_SnCl] by stirring at 80 °C for 1 h, the peak appeared at 34,120 Da for weight average molecular weight (*M*_*w*_) and at 31,640 Da for number average molecular weight (*M*_*n*_) (Figure 6). The ratio of average molecular weights to the number average molecular weights (*M*_*w*_/*M*_*n*_) was 1.07 (Table 1).

**Table 1.** Data for PCL obtained from GPC measurements,

Catalyst (15 mg)	Temperature (°C)	Time (h)	*M*_*w*_ (Da)	*M*_*n*_ (Da)	PDI	% Conversion
[Bu(PFH)_2_SnCl]	60	10	6490	5600	1.03	60
[Bu(PFH)_2_SnCl]	60	17	11,655	11,125	1.05	100
[Bu(PFH)_2_SnCl]	80	1.0	34,120	31,640	1.07	100
[Bu(MAc)_2_SnCl]_2_	80	1.0	6680	6472	1.03	80
[Bu(Piv)_2_SnCl]_2_	80	1.0	21,530	20,460	1.05	100
[Bu(PH)SnCl]	80	1.0	6640	6120	1.08	92
[Bu(Pyd)SnCl]_2_	80	1.0	5050	4370	1.15	70

Note: MAc = Methacrylate, PFH = perfluoroheptanoate, Piv = pivalate, PH = phthalate, Pyd = 2,6-pyridinedicarboxylate.

When compared with other acidic or basic catalysts, these single-site tin catalysts were very effective in the ROP of ε-caprolactone in short times.[29–31] It is also important to note that the direct reactions between BuSnCl_3_ and carboxylic acids instead of K-carboxylates gave the tin compounds including two chloride ligands per tin atom. When these tin compounds were used as catalysts in the polymerization of ε-CL, the higher molecular weight polymers were obtained with the higher ratio of *M*_*w*_/*M*_*n*_ (1.25–1.51) at 80 °C for 17 h as seen in Table 2.

**Table 2.** Data for PCL obtained from GPC measurements.

Catalyst (15 mg)	Temperature (°C)	Time (h)	*M*_*w*_ (Da)	*M*_*n*_ (Da)	PDI	% Conversion
[Bu(MAc)(MacH)SnCl_2_]_2_	80	17	40,050	32,110	1.25	80
[Bu(PFH)(PFHH)SnCl_2_]	80	17	30,800	20,400	1.51	100
[Bu(Piv)(PivH)SnCl]_2_	80	17	15,720	10,950	1.44	100
[Bu(PH)SnCl_2_]	80	17	24,490	21,440	1.44	100
[Bu(Pyd)SnCl_2_]_2_	80	17	18,650	16,200	1.45	85

Note: MAcH = Methacrylic acid, PFHH = Perfluoroheptanoic acid, PivH = Pivalic acid.

DSC analysis was performed to determine the melting point and the degree of crystallinity of PCL. The degree of crystallinity was calclulated according to the method described previously *X*c = Δ*H*_*m*_/$\Delta H_{m}^{o}$.[32] The enthalpy of melting (Δ*H*_*m*_) of the sample, obtained by the measurement of the area under the peak (Figure 7), can be transformed into the relative degree of crystallinity (*X*c) by dividing of the enthalpy of melting by a value of −139*.*3 J g^−1^ for the enthalpy of melting ($\Delta H_{m}^{o}$) of 100% crystalline PCL.[33] Some physical data calculated and obtained from DSC measurements are given in Table 3. PCL pepared with [Bu(PFH)_2_SnCl] melted at 64.78 °C while PCL pepared with [Bu(Piv)_2_SnCl]_2_ melted at 64.04 °C. This small difference was related with the degree of crystallinity. In other words, PCL pepared with [Bu(PFH)_2_SnCl] had somewhat more crystalline than PCL pepared with [Bu(Piv)_2_SnCl]_2_.

**Table 3.** Data for PCL obtained and calculated from DSC measurements.

	Onset temp (°C)	Peak temp (°C)	Endset temp (°C)	Melting heat (J/g)	Degree of crystallinity
PCL prepared with [Bu(PFH)_2_SnCl]	61.40	64.78	67.54	−97.69	70.13
PCL prepared with [Bu(Piv)_2_SnCl]_2_	59.25	64.04	66.99	−94.19	67.62

## 7. Conclusion

In this study, it was shown that carboxylates derivatives were effective ligands for modification of butyltrichlorotin(IV). Five tin carboxylate compounds were prepared, characterized, and used as catalysts for the polymerization of ε-caplolactone at 80 °C and have somewhat different influence on the conversion of ε-caplolactone. The new liquid tin pivalate, methacrylate and perfluoroheptanoate compounds were more active catalysts than solid tin dicarboxylate compounds and other known tin catalysts in the polymerization of ε-CL. The conversion of ε-caprolactone was completed in just 1 h under solvent free condition by tin carboxylates. The structures of polymers were characterized by GPC, DSC, NMR, FTIR, and MS spectrospopies. As seen from ^13^C NMR experimental data, there was just one peak for each carbon atom. There was just one narrow molecular weight distribution peak with ratio of *M*_*w*_/*M*_*n*_ = 1.0 in the gel permeation chromatogram. DSC measurements showed that there is one crystalline phase. All these data were evidence of the regular polymerization of ε-CL and indicating the characteristic of single site tin catalysts.

## Disclosure statement

No potential conflict of interest was reported by the authors.

## Funding

This work was supported by the research foundation of Kocaeli University [project number 17/2014].
